# Filling Teeth

**Published:** 1861-06

**Authors:** W. M. Rogers


### DENTAL REGISTER OF THE WEST.

Vol. XV.] JUNE, 1861. [No. 6.

### Original Essays and Communications.

### FILLING TEETH.

(Read before the Kentucky State Dental Association, April 9, 1861.)

BY DR. W. M. ROGERS.

The filling of decayed teeth is a department of professional duty, in which a greater number of individual operations are performed than in any other, and as each of these efforts involves, in a very great degree, the comfort and happiness of the patient, as well as the character and reputation of the operator, we may safely conclude that any thing which may throw light upon this subject will be of interest to those to whose peculiar office the treatment of these organs appertains.

I would not presume to enter into a general detail of my own manner of filling teeth. I only propose to notice in something of regular order a few things which I conceive to be of importance, gathering in only such corollary details as may sustain an intimate relation to the ultimate success of the operation, and guarding some of the sources which my observation teaches me to believe are causes of failure.

The confidence of the patient is, of course, presumed to be extended to the operator; without this, it were better that nothing should be attempted.

After an examination of the denture, and the decision upon the indications there presented, it is my practice to commence operations, all things being equal, upon the molars in preference to the remaining teeth, and then upon the bicuspids, taking the cuspidati and incisors last under treatment. This course is better calculated to secure the attendance of the patient, until the whole denture has received attention, as few persons once aroused to the necessity of submitting to the hands of the dentist, will desist at any stage short of the security of the front teeth.

With inexperienced or very timid patients, it may be proper to commence upon the simpler and less painful cavities, progressing to those involving more complication, and of a higher degree of sensitiveness, thus familiarizing the subject for the "tug of war."

Prior to the preparation of a cavity for filling, it becomes necessary to secure such an approach as will permit the proper manipulations, with instruments adapted to the purpose of excavating the decay, and of introducing, consolidating and finishing the filling.

If the defect is upon the approximal surface of the tooth, one of the ordinary methods of separation is resorted to, as circumstances may seem to demand.

For separations made by cutting away the surface of the tooth, I prefer the chisel, or rather I should say, the hatchet, applied somewhat after the manner of the chisel. This instrument has a blade about one half an inch long, the edge of which is about three-eighths of an inch broad, its plane extending on a line with the shaft, at an angle of about sixty degrees therefrom.

I have preferred this to the chisel, for the reason that in manipulating with it the thumb of the hand in which it is held may, in most cases, rest upon an adjoining tooth, guiding the course of the blade, and giving security from accidents.

It may be used upon the lingual margins of the anterior teeth with great facility.

For the separation of the posterior molars, a similar instrument, but having the line of the blade transverse to the shaft, may be used with facility.

A separation once being resolved upon, every consideration urges the importance of making it wide enough to secure the end contemplated, in the most complete manner. *Space* in which to manipulate *properly* is an absolute requisite. The character of each separation must be determined by the conditions of the individual case.

For separations by mechanical pressure, I prefer to use caoutchouc; intervening it between the affected teeth. A separation to any extent once being secured by this means, should the teeth become irritable and painful, it is well to desist for the time, yet being careful to retain the space already made by the substitution of a piece of cork, or other less elastic substance, in place of the gum. When the unfavorable symptoms subside, the treatment with the gum may be resumed. With this precaution, cases may be urged to a successful result, that the operator might otherwise be tempted to give up in despair.

In the preparation of the cavity of decay, the manipulations must, of course, vary with the almost endless variety of conditions presented for treatment.

I have, after careful observation, become satisfied that, as a general principle, it is better to have a cavity when finished something larger within than at the orifice. Although such an excavation may induce a necessity for precautions in filling, not applying to one having parallel walls, yet when the work is once done, and properly done, I think there will be greater security in the filling.

For the purpose of embodying as far as possible in a small space that which might otherwise require a very prolix explanation, I propose to take a condition of decay often found upon the upper molars, and proceed to the manner of its treatment.

We will suppose the tooth to have a central crown cavity, with lines of decay radiating along the approach of the enamel as it ascends to the depressions between the cusps. The line extending to the posterior terminates in a fissure affected by decay, and passing from the buccal angle across the coronal surface, over the lingual angle, and thence upon the lingual surface, terminating in an enlargement about its center.

In preparing this for filling, I would first break away the margin of enamel over the central cavity, giving symmetry to the periphery by the use of the burr drill. Then enter a suitable drill upon the extremity of one of the radiating lines, and drill thence in the direction of the line until the decay is eradicated, or otherwise enter the drill at any number of points upon the affected line to a proper depth; then with a suitable instrument cut away the intervening parts, making the elongated cavity continuous throughout all its margins.

It may be found that one or more of the radiating extensions are broader at their emanation from the central cavity, and converge to a point at the extremity; in such case, I would enter the drill at the point, and then cut away the parts intervening between this and the center with broad bladed excavators. The same principles of procedure may be applied in the preparation of the transverse fissure. That part of this latter cavity which forms the connection by its extension over the angle, with the defect upon the lingual surface, often proves a source of great annoyance in its preparation. There is danger that an excavator or drill applied here with effective force may slip and wound the mouth.

Here a separating file, secured in a suitable carrier, may be used to great advantage, by filing up through the fissure, being careful at the same time to keep the plane of the file on a line continuous both with the coronal and lingual decay.

It is advisable to pare away the sharp edge of the margins of the cavity throughout its whole extent. This is a matter of greater importance in friable teeth, for the reason that a very delicate point may be abraded by the pressure necessary to the consolidation of the filling, when an obtunded margin will not be thus injured. Such imperfection will, of course, invite a recurrence of decay.

In preparing the gold for filling, I would proceed as follows: Cut the sheet into strips of one-third to three-fourths of an inch in width, and fold these longitudinally. When folded, the width of the slip should be a little greater than the depth of the cavity for which it is designed. This is then rolled into cylinders upon the point of a small broach, making one or several of these from each slip as may be desirable.

Then prepare a few pieces by folding them flat upon a watch spring; the length and breadth of these flat pellets should correspond to the purposes to which they are to be appropriated, as hereafter described.

In addition to this, have at hand some of these slips folded as at first directed, and cut into two or three pieces.

The gold should be handled with great care, using the forceps in lifting it, and in every way avoiding as much as possible its contact with the fingers. When ready for the filling, it should be soft, neat and smooth. With every thing thus prepared, the cavity having been syringed and dried, and the encroachments of the saliva being guarded against, it will be proper to commence the operation of filling by introducing one of the larger cylinders into the central cavity, handling it very softly with the forceps, and thrusting it fully to the apex of the cavity, yet permitting the lower end to protrude. This should then be pressed rather gently to one of the walls, when another cylinder should, like the first, be placed carefully in position with as little disturbance of its shape as possible—this is also forced with gentle pressure to the walls. Thus cylinder after cylinder is introduced, until the outer wall is lined at all points with the gold, which will now remain "in situ," while attention is given to other parts of the operation.

With the forceps now introduce one of the flat pellets before prepared into either of the radiating lines, having it to extend the whole length of the excavation, from the extremity to the central filling. If the cavity will permit, this may be placed to one side with slight compression, and another introduced by its side, when a flat pointed instrument should be entered between the two, and the space thus produced filled with the slips prepared for that purpose; then again introduce the flat point and fill as before, repeating this to the complete consolidation of the gold. If the cavity will only admit one pellet, this may be of sufficient width to permit its central part to be forced up along the whole line of its extent, and immediately consolidated—or if advisable, steps may be used in its opened center, and afterwards be compacted by direct pressure.

The same process is applicable to all the radiating cavities, as also in many cases, to the posterior transverse continuation.

After filling the radiating lines, it will be necessary to recur to the unfinished central portion. Into this introduce a small, round-pointed instrument; when withdrawn, follow by a larger, and again by a yet larger and larger one, until the gold is as thoroughly compacted as the integrity of the tooth will permit. We have then a cavity within the filling. This should be filled as if a simple excavation, until a sharp plugger can not be entered by any ordinary effort.

It frequently occurs that there is an enlargement at the juncture of the posterior radiation with the transverse cavity. In such case, after introducing the flat pellets into the transverse cavity, cylinders may be used to fill this enlargement, a procedure which adds to the strength of the filling, as this point usually rises higher into the dentine, and consequently gives better surface for attachment. The same remarks apply to the enlargement at the lingual extremity of the continuation of this cavity.

The pellets placed in the coronal transverse cavity should protrude full out at the angle of the tooth, and those in the lingual extension should reach the whole remaining length of that cavity; or better yet, the pellets may be placed in the coronal and lingual parts alternately, intersecting each other at the angle. If neither of these processes is practicable, then, after filling the two extremities, without reference to each other, again introduce the file as before directed in excavating, cutting up into the gold, until the file reaches the line to which it was at first carried. This results in a simple incision, one end of which is upon the coronal, and the other upon the lingual surface. This may be filled by pellets, extending throughout its length, and then by strips, as before directed for other parts of the filling.

I think these elongated pellets of importance, because the gold is less liable to abrade at the angle of the tooth, or to be dislodged by any accidental violence, than by any other mode, having less of attachment throughout the extent of the cavity.

The gold being introduced and consolidated, it now remains to finish the filling, by filing away all superfluity even with the margins; when the work may be polished by the use of stones, powders, etc., as the operator may prefer.

This cavity being disposed of, I propose to consider a condition often presented upon the bicuspids, although not by any means peculiar to them.

It is that of an approximal decay complicated with a supervening defect of the coronal surface. In this case, the file or chisel may be used very sparingly by cutting away the coronal surface of enamel which lies over the approximal decay, thus throwing both cavities into one. This excavation when finished, should be larger within than at the approximal opening. The cervical wall should also be a little deeper within than at the margin, but if deeper, it is well to emphasize it, only a little deeper.

In introducing the gold, place as large a cylinder as can be used without marring its integrity as soft gold, in the cavity, the end protruding out from the approximal orifice, and with a flat-bladed plugger press it gently to one of the side walls; apply another cylinder in the same manner to the opposite wall, then introduce a third between these—this should be large enough to fill the intervening space from side to side, and yet not so large as to drag the first two from their positions as it is carried to its place against the cervical wall. This being in place, with a square or oblong pointed plugger, of a size at the point that just permits it to pass between the side cylinders, press the intervening gold with sufficient force to the cervical wall to consolidate it—this, when properly done, will secure the side cylinders in place. If these last have been chosen of sufficient size to protrude from the cavity at the coronal opening after the lateral pressure has been applied, it will only remain to introduce another and another cylinder between these, until the cavity is full, successively consolidating each one thus placed before another is introduced. Or otherwise the space between the side cylinders, remaining after the first consolidation of intervening gold, may be treated as a primary cavity, using of course in this smaller space cylinders adapted to the case—such as those first used would here be too large. But if the side gold does not protrude from the coronal opening, then all the gold first introduced should be consolidated as a simple filling,—after, perhaps, the prior introduction of one or two cylinders in the center, according as the depth of the cavity may require or the length of the side gold will permit. The remaining coronal extremity may be filled by the same process.

A case similar to this is described in the Dental Cosmos for January, 1860, by Dr. Charles Woodnutt, the object of which is to avoid the use of the file in the treatment of approximal cavities, by cutting down the coronal surface into the cavity. In either event, I would prefer to separate to some extent, as there generally exists some superficial imperfection, which should be removed, and the approximal margins of the filling are all the safer for optical examination. The grand object, however, to which I have desired to invite attention is rather to the *filling* of the tooth.

Another case will complete the individual cavities of which I design to speak. We will now take a central incisor decayed upon one of its approximal surfaces, having a considerable part of its lingual wall broken away, leaving a very narrow point upon which to operate near the delicate cutting edge of the tooth—this will not afford a wall as a reliable antagonist to the cervical margin. With such a condition of decay, I would proceed to cut as deep into the tooth as the safety of the nerve will permit, excavating forward to the labial and backward toward the lingual surfaces at the same time, inclining the cervical wall upward as it penetrates into the tooth.

This cervical part of the cavity will then be enlarged within the walls, meeting each other rather angularly. Now secure at the point of the tooth as good a wall as the conditions will admit. This done, the cavity will present a V shaped orifice, the base lying upon the cervical wall, and the apex extending toward the cutting edge of the tooth. The margins of the orifice upon the lingual line yet remain, of course, imperfect.

In introducing the gold, place a large soft cylinder so as to occupy a position both against the cervical and the lingual walls; this, when pressed into the angle formed by the approach of these walls, should protrude below the broken lingual margin, reaching thence to the opposite side of the fracture ; the end of the cylinder should likewise appear full out from the approximal orifice. The plane of the inner surface of the flattened gold should be about parallel with the labial wall. The gold being in this condition, introduce as large a cylinder as may be used between the gold in the tooth and the opposing surface; press this up against the cervical wall, and consolidate well. It may be expedient to place two or more smaller, instead of this larger cylinder last mentioned; if so, arrange them regularly side by side in the cavity, and then consolidate. This will secure the gold at that point with firmness. In the same manner place other cylinders, arranging them carefully, and consolidating well as the work progresses.

When the point is reached at which the consolidation of gold in the direction of the axis of the tooth must cease for want of a lingual wall to resist the filling on that side, it will become necessary to resort to the imperfect excavation at the cutting edge, for additional security to the remainder of the filling.

When brought to this stage, the secondary cavity now remaining will appear about as follows: We have, first, the labial plate entire; a small part only of the lingual wall, the fractured part being to some extent supplied by the extension of the first cylinder introduced. Again, we have the excavation at the point, and opposite to this the wall of consolidated gold, the face of which should incline a little forward to the labial plate of enamel and ascend a little upward as it extends into the tooth. This cavity may now be filled with slips or cylinders, applying the pressure of the final consolidation outwardly and obliquely in the direction of the excavation. This last case is one of those in which crystal gold would seem to be especially applicable.

One of the main principles which I have attempted to illustrate in the preceding cases is that of giving strength to the weaker points of a filling, by continuing the gold there applied from such portions of the cavity as will secure the firmest attachment which the case may permit.

A very important requisite to the perfection of a filling is good foil. Every case does not of necessity require a material possessing the same uniform qualities. Gold capable of being worked into a very excellent coronal filling may be totally unfitted for the same result in an approximal cavity of difficult access.

The properties of foil to which these different results are referrable are its degree of softness and of adhesiveness. Purity and toughness are absolute prerequisites in any case. The softer the gold when first introduced, the better may it be adapted to the minute irregularities of the cavity. It avails little that the gold be soft when it comes into the hands of the dentist, if in the manipulations preparatory to its application to the purposes of a filling, it be frequently and rudely handled.

The advantages of foil over gold in mass, for the purposes of a filling, lie in the fact that the laminæ of foil may be made to lie loosely upon each other, and yet be brought into a condition of comparative solidity by the application of pressure, under which application they may slide upon each other, thus admitting adaptation to minor irregularities among their own surfaces, as well as to those of the cavity in which the gold is placed.

If this statement is true as a primary fact, then the superiority of thin to thick foil for general purposes in dentistry follows as a consequence; and hence also, a given weight of No. 4 foil, prepared for a filling, being loose in its structure, is susceptible of a greater comparative compressibility than the same weight of No. 20, prepared in like manner—a difference of great importance, as that which yields most kindly to the impress of the instrument, upon the one hand, takes shape to the opposing cavity with like readiness upon the other.

In proportion as foil is possessed of the property of adhesiveness, so will it approximate to the stubbornness of massive gold, as its laminæ are brought in contact under pressure. This property renders it necessary that the application of pressure should be made with the greater caution at the right time, in the right direction, and of the proper degree, for the security of the immediate purpose.

The softer and more friable the structure of the tooth, the greater is the necessity for the softer gold, for with this there is less danger of abrading the margins of a cavity, if a *sufficient* pressure *only* has been used to secure proper consolidation. It will not be presuming too much to say, that very many otherwise good fillings are ruined for their purposes, by inattention to this point. The abrasion of a margin may not at first appear, but time will certainly develop the imperfection.

In the course of this paper, I have indicated a preference for the slight enlargement of the interior of cavities in preparation for filling. This preference is founded upon an idea that in addition to the many causes which conspire to the destruction of fillings in the teeth, there is one not generally noted, to which I ascribe importance.

With some hesitation I assert it that the difference in the expansibility of dentine and enamel from that of gold, under the varying degrees of caloric to which they are subject in the mouth, considered in connection with the peculiar condition in which most of our operations in filling teeth are discharged from our hands, has a *tendency*, by philosophical necessity, to expel the filling from the cavity during a long process of the operation of these causes. An object held on a strain between two points tends to slide from the grasp of the weaker point to that of the stronger.

I think it would be found to be a fact upon investigation, that by far the larger number of our fillings are more thoroughly consolidated at the surface than in the interior, and that as we ascend deeper into a filling, the less of density we find.

Without being committed to the confession that there is any necessity that such should be the case, it is only pertinent to the present purpose to assert that the fact so exists.

In the thousands of times in which the temperature of the teeth and the fillings in them is altered in the mouth, the gold in all such cases as these just mentioned, in undergoing contraction, goes from the point of less adhesion—from the direction of the interior—to that of greater consolidation, and consequently of greater security, in the direction of the surface. It is true that if we suppose, for the moment, that the gold is undergoing expansion from an elevation of temperature, then it may be elongated in the direction of the lesser resistance and toward the interior of the tooth; yet the resisting surfaces to inward extension are co-extensive with the walls of the cavity, and are always in operation, while any very minute length of the filling, once protruded from the orifice, ceases forever to take part in the action, except it may be made potent in resisting any inward retraction, by virtue of a very slight enlargement on being relieved from the grasp of the orifice. In forming the cavity larger within than at the orifice, I would purpose to remedy this supposed evil. More or less inequality exists in the interior of almost every cavity—a happy fortuity if what I have said is correct. It will at once be perceived that these intero-enlarged cavities will require especial attention, to the proper consolidation of the interior part of the filling.

Another remark upon the manner of placing gold in a cavity, and I am done. All that has been said is upon the presumption that the gold is introduced in such manner that the laminæ shall extend from the bottom of the cavity outwardly to the margin. These should extend *directly* out and on as nearly parallel lines as possible, and should have as much lateral consolidation as can be safely given prior to any attempt to consolidate in the direction of the axis of the filling, otherwise the gold when polished down will present upon its surface many points at which the ends have been pressed below the disc, and these being severed from their actual connection with the laminæ, will in time lose their attachments, thus spoiling the beauty of the filling. In proportion, however, as the gold is adhesive, will this result be lessened.

Having marked out a specific course to follow, which will in itself consume too much, perhaps, of the time of the association, I have not spoken of many points of interest to myself, and must claim credit for at least this exhibition of discretion.

